# Tuberculosis Infection among Young Nursing Trainees in South India

**DOI:** 10.1371/journal.pone.0010408

**Published:** 2010-04-29

**Authors:** Devasahayam J. Christopher, Peter Daley, Lois Armstrong, Prince James, Richa Gupta, Beulah Premkumar, Joy Sarojini Michael, Vedha Radha, Alice Zwerling, Ian Schiller, Nandini Dendukuri, Madhukar Pai

**Affiliations:** 1 Department of Pulmonary Medicine, Christian Medical College, Vellore, Tamil Nadu, India; 2 Department of Medicine (Unit I), Christian Medical College, Vellore, Tamil Nadu, India; 3 College of Nursing, Christian Medical College, Vellore, Tamil Nadu, India; 4 Department of Microbiology, Christian Medical College, Vellore, Tamil Nadu, India; 5 Department of Epidemiology and Biostatistics, McGill University, Montreal, Canada; 6 Division of Clinical Epidemiology, McGill University Health Center, Montreal, Canada; 7 Respiratory Epidemiology & Clinical Research Unit, Montreal Chest Institute, Montreal, Canada; National Institute for Infectious Diseases L. Spallanzani, Italy

## Abstract

**Background:**

Among healthcare workers in developing countries, nurses spend a large amount of time in direct contact with tuberculosis (TB) patients, and are at high risk for acquisition of TB infection and disease. To better understand the epidemiology of nosocomial TB among nurses, we recruited a cohort of young nursing trainees at Christian Medical College, a large, tertiary medical school hospital in Southern India.

**Methodology/Principal Findings:**

Among 535 nursing students enrolled in 2007, 468 gave consent to participate, and 436 underwent two-step tuberculin skin testing (TST). A majority (95%) were females, and almost 80% were under 22 years of age. Detailed TB exposure information was obtained using interviews and clinical log books. Prevalence of latent TB infection (LTBI) was estimated using Bayesian latent class analyses (LCA). Logistic regression analyses were done to determine the association between LTBI prevalence and TB exposure and risk factors. 219 of 436 students (50.2%, 95% CI: 45.4–55.0) were TST positive using the 10 mm or greater cut-off. Based on the LCA, the prevalence of LTBI was 47.8% (95% credible interval 17.8% to 65.6%). In the multivariate analysis, TST positivity was strongly associated with time spent in health care, after adjusting for age at entry into healthcare.

**Conclusions:**

Our study showed a high prevalence of LTBI even in young nursing trainees. With the recent TB infection control (TBIC) policy guidance from the World Health Organization as the reference, Indian healthcare providers and the Indian Revised National TB Control Programme will need to implement TBIC interventions, and enhance capacity for TBIC at the country level. Young trainees and nurses, in particular, will need to be targeted for TBIC interventions.

## Introduction

The risk of transmission of *Mycobacterium tuberculosis* (TB) between patients and health care workers (HCWs) is well recognized [1], [2], [3]. Until recently, TB infection control was neglected in TB endemic countries [4]. However, with the emergence of extensively drug resistant (XDR) TB, and the evidence that XDR-TB may be transmitted between patients and staff within the hospital setting, there has been a renewed interest in TB infection control (TBIC), especially in resource limited settings with high TB and HIV prevalence [5], [6].

Several TBIC interventions have been proposed for high income countries [7], but many of these cannot realistically be implemented in low income settings [4]. There is also lack of strong evidence on the efficacy of TB infection control interventions in high incidence settings [8]. Acknowledging these realities, the Stop TB Partnership created a subgroup on TBIC, and the World Health Organization (WHO) released a guideline for TBIC in resource limited settings in 2009 [8].

HCWs in high TB burden countries have a higher risk of TB infection and disease as compared to the general population because of their exposure to large numbers of recognized and unrecognized smear positive pulmonary TB cases managed at the hospital, and due to inadequate implementation of TB infection control. The estimated prevalence of latent TB infection (LTBI) among HCWs in low and middle income countries is 54%, with an annual risk of TB infection (ARTI) ranging from 0.5 to 14.3%[1]. The median annual incidence of TB infection in low and middle income countries attributable to health care work has been estimated at 5.8%[1].

Recent data from India suggests that nearly 40% of HCWs may have LTBI, as measured by positivity in either the tuberculin skin test (TST) or interferon gamma release assay (IGRA), and increasing age and years in the health profession were significant risk factors for positivity [9]. The ARTI among medical and nursing trainees has been estimated to be approximaely 5% [10], which is substantially higher than the ARTI in the general population (estimated at 1.5%) [11]. Consequently, the incidence of active TB in HCWs in India is substantially higher than that in the general population [12].

Among HCWs, nurses spend more time in contact with infectious TB patients, and are at high risk for acquisition of TB infection and disease [1]. A systematic review showed that prevalence of LTBI among nurses ranged from 43% to 87%, and in almost all studies, the prevalence of LTBI in nurses was 1.3% to 35.6% higher than in other HCWs [1]. Recent studies from India [9], [10], Brazil [13] and Zimbabwe [14] have reported very high rates of LTBI and disease in nursing trainees, and this may be due to intense and repeated exposure to TB patients during clinical apprenticeship. Recently, our group reported the tragic loss of the life a young nurse to XDR-TB. This is the first reported XDR-TB death among HCWs in India [15], and underlines the problem of nosocomial TB, and the risk nurses face from this neglected problem.

To better understand the epidemiology of nosocomial TB in nursing trainees and to facilitate TBIC policy implementation in India, we have initiated a cohort study among nursing students in a large hospital to assess prevalence and incidence of LTBI. This cohort is larger and more homogenous than similar studies published earlier [9], [12]. Because it includes students in every year of the training program, it allows us to study the increase in risk over time since entering the health care environment. We have also collected extensive information on TB exposure factors, allowing us to examine the relationship between these variables and LTBI prevalence. A better understanding of nosocomial TB will enable targeted TBIC interventions, especially since resources are limited. In this first report, we describe the cohort and present the results of baseline testing.

## Methods

### Study design

Our study was conducted at the Christian Medical College (CMC), Vellore, a large (2200 beds) tertiary referral medical school in Vellore, a town in Southern India. We prospectively approached all enrolled nursing students for participation in a cohort study, to assess LTBI prevalence annually over several years. The study protocol was approved by the institutional review boards at both CMC, Vellore and McGill University, Montreal.

### Setting

The district of Vellore has an annual TB case detection rate of 148/100,000 [16]. A previous report described 126 cases of active TB among various health care workers at CMC over a 10 year period, but the prevalence of LTBI among nursing students was not known [17]. Hospital infection control policy states the adult patients with smear positive pulmonary TB are to be placed in a single room with closed doors and open windows, and surgical masks are to be worn by staff entering the room. Due to constraints of space and resources, this policy is not widely implemented. Only one negative pressure room is available, and N95 respirators are not routinely used. There is no policy for routine annual TST screening of all HCWs.

### Study participants

The College of Nursing at CMC offers several different training programs (Diploma, BSc, Post Diploma courses, Fellowship courses, MSc, Doctoral [PhD] programs) and, on average, 500–600 students are on training at any given time. The diploma (3 year program) and BSc (4 year program) are undergraduate programs, while post-diploma, MSc and PhD are considered post-graduate programs. All students enrolled in 2007 for the various programs were approached for consent to participate in the study, first by notification through a large meeting, then in smaller groups according to training courses. Consenting students signed a written consent form. Students under the age of 18 years were required to obtain written parental consent as well as provide an assent. Students with past history of active TB were excluded from the study.

### Measurement of exposure to TB and other patient characteristics

Undergraduate nursing students at CMC are required to maintain detailed log books of their clinical rotations and activities. Details of TB exposure during training were obtained from individual clinical log books. For senior nursing students in the third year of the diploma program and for those in the Post B.Sc. and M.Sc. programs, details of TB exposure were based on recall (because they were not required to maintain logs). The log books document specific numbers of patients with TB cared for, and days of clinical experience on high risk areas. Isolation ward and DOTS clinic were defined as high risk wards since they consistently care for TB patients. Medium risk areas were defined as those with discharge records containing greater than 15 cases of active TB within one year; this included general and subspecialty medical wards, cardiothoracic surgery, intensive care, emergency and medical outpatients. All other work areas were classified as low risk for TB exposure.

Students completed a written case report form, providing information on demographics, socio-economic and educational status, previous work in health care, specific details about ward rotation on high or medium risk wards, patient-days caring for patients with active TB (number of patients multiplied by number of days of care), known contact with smear positive TB cases in the community or hospital setting, past history of TB, a symptom screen for current active TB, and an assessment of any immunocompromising condition or treatment. This was reviewed by a physician over a personal interview, during which the participants were screened for any current TB symptoms and referred for physical examination, if required. An examination including BCG scar inspection, height and weight were also performed. On account of the anticipated low prevalence of HIV in this young, healthy cohort, we did not perform HIV testing.

### TST testing

Students were tested with TST at baseline. TST was placed in a two-step protocol [18]; all students with initial negative TST underwent repeat TST testing at 7–14 days to determine boosting, because of the anticipated need for repeat testing. Two Tuberculin Units (0.1 ml) of RT23 PPD (Staten Serum Institut, Copenhagen) was injected intradermally into the volar aspect of the left forearm. After 48–72 hours, the maximum diameter of palpable induration was measured by a trained TST reader. TST was considered positive if induration was ≥10mm (after the two-step protocol). Any adverse events were recorded.

### Management of symptoms and TST positives

If students screened positive for symptoms of active pulmonary TB, they were referred for investigation for active TB with three sputum smears and a chest x-ray. Any student diagnosed with active TB was treated with standard first line chemotherapy as per national guidelines, using a DOTS strategy. Students were not routinely offered isoniazid preventive therapy (IPT) if they were diagnosed with LTBI. IPT is not routinely offered to HCWs with LTBI in India because of the very high background prevalence of LTBI, the risk of repeated exposure and reinfection following therapy, the risk of drug toxicity, concern about widespread INH mono drug resistance, and the lack of strong evidence that preventive therapy has long-term efficacy in populations that are repeatedly exposed to TB. However, the protocol ensured that all students diagnosed to have TST conversions during follow up will be offered preventive therapy.

### Statistical analysis

Descriptive statistics were used to summarize the distribution of demographic and clinical variables, as well as variables measuring exposure to TB. A histogram was used to examine the distribution of continuous TST reactions. We used univariate logistic regression to evaluate the risk of a positive TST result associated with demographic, clinical and TB exposure variables. Increasing age is a well known predictor of TB risk. In order to separate the effect of age from the effect of time spent in health care, we defined the ‘age at entry’ into healthcare as the difference between these two variables. We hypothesized that the age of entry captures the cumulative effect of exposure prior to being in a health care environment (i.e. community exposure), while the number of years spent in health care after entry captures the effect of nosocomial exposure in the health care environment (either as a trainee in a hospital or during hospital work). For example, if a 20 year old student joined the BSc program when she was 18 years old, age at entry into healthcare would be 18 years and years spent in healthcare after entry would be 2 years.

We fit a multivariate logistic regression model for predicting positive TST based on age at entry, time spent in health care after entry, sex, BCG scar and contact with TB patients. We did not include variables such as ‘worked prior to the training program’ and ‘days caring for pulmonary TB patients’ as these were highly correlated with the number of years in health care. We also examined the linear correlation between continuous TST and continuous measures of exposure to TB.

We estimated LTBI prevalence using the Bayesian latent class analysis (LCA), a method that allowed us to adjust the observed TST results based on our prior knowledge of TST performance characteristics [19]. As described previously, the LCA is a more realistic model-based approach to estimating prevalence in the absence of a gold-standard test for LTBI. Despite being the conventional test, TST is known to have imperfect sensitivity and specificity. However, because a lot is known about the performance of TST in various populations, we can use this prior knowledge to adjust the observed data. We based our prior information on the results of recently published meta-analyses [20], [21], [22], and assumed the range of sensitivity and specificity of TST to be 70%–90% and 60%–95%, respectively. Thus, the probability of a positive TST test is likely to be an overestimate of the prevalence. Bayesian latent class analysis adjusts the observed TST-based prevalence using our prior knowledge of TST sensitivity and specificity.

LCA is based on the notion that the observed results of various imperfect tests for the same disease are influenced by a common, underlying latent (unobserved) variable, the true disease status [19]. Increasing the number of imperfect tests and/or taking into account prior knowledge, increases our knowledge of the latent disease status. One increasingly used medical application of LCA is evaluation of diagnostic tests in the absence of a gold standard. The latent class analysis was carried out using WinBUGS [MRC Biostatistics Unit, Cambridge, UK], following the method we have previously described in detail [19]. The remaining analyses were carried out using SAS [SAS Institute Inc, Cary, NC, USA].

## Results

### Description of the cohort

**Figure 1:**
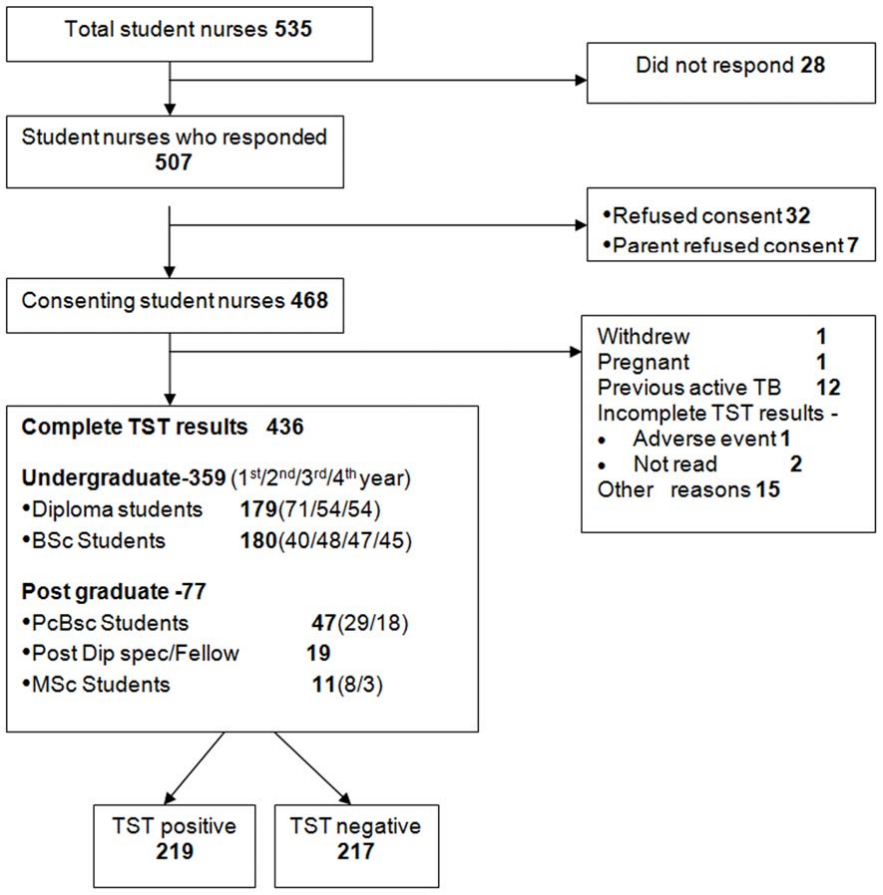
Study flow chart.

Figure 1 shows the study recruitment flow chart. Among 535 nursing students enrolled in the College of Nursing in 2007, 468 gave consent to participate, and 436 (82% of total population) provided complete data including valid TST results. 95% were females, and almost 80% were under 22 years of age. Diploma and BSc course trainees represented 82% of students. 74% had a documented BCG scar. There were no known HIV-infected participants. Tables 1 and 2 provide more information on the baseline characteristics of the cohort. The linear correlation between age and the number of years in health care was 0.965 (95% confidence interval (CI): 0.958, 0.971). The correlation between the age at entry and the number of years in health care was lower, 0.36 (95% CI: 0.28, 0.44), though it was significantly different from zero.

**Table 1:** Descriptive and demographic characteristics of the participants (N = 436).

Variable	N	%
Total number of participants in the study	436	100
**Age categories (years)**		
17	52	11.93
18	100	22.94
19	96	22.02
20–21	95	21.79
> = 22	93	21.3
**Age at entry (years) (Age - years in healthcare)**		
<18	343	78.67
> = 18	93	21.33
**Sex**		
Female	412	94.50
Male	24	5.50
**Body mass index (kg/m^2^)**		
< = 19	107	24.54
>19	329	75.46
**Highest education achieved prior to joining the nursing training at CMC hospital**		
Class 12	356	81.70
Diploma	58	13.30
Bachelor	21	4.80
Masters	1	0.20
**Current nursing course**		
Diploma, year 1	71	16.28
Diploma, year 2	54	12.39
Diploma, year 3	54	12.39
BSc, year 1	40	9.17
BSc, year 2	48	11.01
BSc, year 3	47	10.78
BSc, year 4	45	10.32
Post-diploma, post-BSc and MSc	77	17.66
**Average Household Monthly Income (Indian Rupees)**		
Less than 5,000	130	29.82
5,000–10,000	155	35.55
10,001–20,000	79	18.12
20,001–30,000	27	6.19
More than 30,000	30	6.88
Refused to provide income data	15	3.44

LTBI: Latent Tuberculosis Infection; TST: Tuberculin Skin Test; TB Tuberculosis.

**Table 2:** Clinical characteristics of the participants (N = 436).

Variable	N	%
Total number of participants in the study	436	100
**BCG scar**		
Absent	96	22.02
Present	320	73.39
Uncertain	20	4.59
**Past history of tuberculin testing**		
No	395	90.60
Yes	32	7.34
Unknown	9	2.06
**Previous TB assessment (i.e. worked-up for TB disease in the past)**		
No	401	91.97
Yes	29	6.65
Unknown	6	1.38
**Current symptoms meet criteria for referral to rule out active TB**		
No	412	94.71
Yes	22	5.06
Unknown	1	0.23
**History of HIV testing in the past**		
No	354	81.19
Yes	82	18.81

LTBI: Latent Tuberculosis Infection; TST: Tuberculin Skin Test; TB Tuberculosis.

### TB exposure and risk factors

Exposure to TB was divided into exposure prior to current training program and exposure during current training program (Table 3). Eighteen percent of students had worked or trained in health care prior to their current training, for a total median health care work of between 1 and 3 years. Prior to testing, sixteen students (4%) had shared a room with a patient with active TB, 27 (6%) had shared a house with a patient with active TB, 11 (3%) had worked with a staff member with active TB, 18 (4%) had attended class with student with active TB and 23 (5%) had socialized with an individual with active TB. During training, 273 (63%) recalled conversational distance contact with a pulmonary TB patient, and 208 (48%) were aware that the contact was smear positive.

**Table 3:** Prevalence of TB exposure and risks factors (N = 436).

	N	%
Total number of participants in the study	436	100
**TB exposure prior to current training program**		
Worked in health care prior to current training program		
No	356	81.65
Yes	80	18.35
**Social contact with a TB patient (previously or during current training)**		
Shared a room with a TB patient		
No	411	94.27
Yes	16	3.67
Unknown	9	2.06
Shared a house with a TB patient		
No	405	92.89
Yes	27	6.19
Unknown	4	0.92
Worked with staff who had TB disease		
No	396	90.83
Yes	11	2.52
Unknown	29	6.65
Attended class with trainee who had TB disease		
No	383	87.84
Yes	18	4.13
Unknown	35	8.03
Socialized with a TB patient		
No	369	84.63
Yes	23	5.28
Unknown	44	10.09
**TB exposure during current training program**		
Source of exposure information		
Log book	305	69.95
Partial data available from Log book	54	12.39
Only recall (no log book)	77	17.66
Conversational distance contact with a pulmonary TB patient		
No	116	26.61
Yes	200	45.87
Yes but don't know if smear-positive	73	16.74
Unknown	47	10.78
Time (years) spent in health care work after entry		
< = 1	110	25.23
>1 but < = 3	203	46.56
>3 but < = 5	46	10.55
>5	77	17.66
Number of days spent working in high risk wards during training program (mean and sd)	5.17	7.13
Number of weeks spent working in medium risk wards	81.92	69.18
Patient-days caring for active TB (mean and sd)	27.40	136.34
Months spent in health care work to date (mean and sd)	35.97	47.43

Of the 436 trainees, 359 students maintained log books and thus details of TB exposure in CMC hospital could be obtained from logs. However, before joining their current nursing training at CMC hospital, 77 students had a history of working in other hospitals, where they did not have to maintain any logs. Thus, in these 77 students, details of TB exposure in the hospital were obtained purely on the basis of recall. Students who used purely recall instead of log books were more likely to be TST positive (OR 4.47, 95% C.I. 2.52, 7.94). Students, on average, spent a mean of 27 (SD 136) patient-days caring for patients with active TB.

### TST results

**Figure 2:**
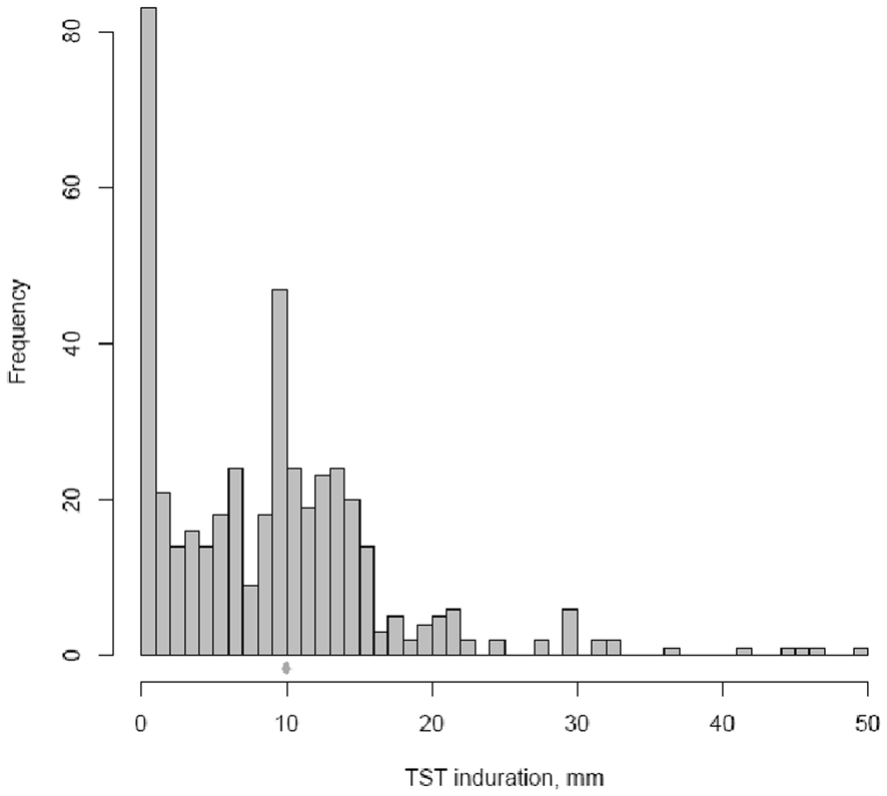
Distribution of tuberculin skin test reactions (induration in mm).

Figure 2 shows the continuous distribution of TST reactions. 71 of 436 students (16%) had no measurable induration. The rest of the distribution was centered around 10 mm (mean 9.53, SD 8.58) with some digit preference for the value of 10 mm. After the two-step TST protocol, 219 students (50.2%, 95% CI: 45.4–55.0) were positive using the 10 mm or greater cut-off, and 82 (18.8%) using the 15mm or greater cut-off. Tables 4 and 5 present univariate odds ratios measuring the association between demographic and exposure variables and TST positivity. Age at entry greater than or equal to 18 (OR 1.77, 95% C.I. 1.11–2.83), age category greater than or equal to 22 years (OR 3.04, 95% C.I. 1.85–4.98), body mass index greater than 19 (OR 1.90, 95% C.I. 1.22–2.96) and higher levels of previous education (OR for diploma 3.14, 95% C.I. 1.7–5.81, OR for university 3.88, 95% C.I. 1.52–9.96) were associated with TST positivity. Presence of BCG scar showed a trend to association (OR 1.52; 95% C.I. 0.96–2.41).

**Table 4:** Association between demographic variables and TST positivity: Results of univariate logistic regression.

Variable	N	TST > = 10mm	
		Positive n (%)	Odds ratio (95% CI)
**Age (years)**			
17	52	25 (48.1)	1 (-)
18	100	44 (44.0)	0.85 (0.43, 1.66)
19	96	45 (46.9)	0.95 (0.49, 1.87)
20–21	95	39 (41.1)	0.75 (0.38, 1.49)
> = 22	93	66 (70.9)	3.04 (1.85, 4.98)
**Age at entry (years) (Age - years in health care)**			
<18	343	162 (47.2)	1 (-)
> = 18	93	57 (61.3)	1.77 (1.11, 2.83)
**Sex**			
Male	24	14 (58.3)	1 (-)
Female	412	205 (49.8)	0.71 (0.31, 1.63)
**Body mass index (kg/m^2^)**			
< = 19	107	41 (38.3)	1 (-)
>19	329	178 (54.1)	1.90 (1.22 2.96)
**Highest education achieved**			
Class 12	354	159 (44.9)	1 (-)
Diploma	57	41 (71.9)	3.14 (1.7, 5.81)
University level	25	19 (76.0)	3.88 (1.52, 9.96)
**Current nursing course**			
Diploma, year 1	71	33 (46.5)	1 (-)
Diploma, year 2	54	19 (35.2)	0.38 (0.13, 1.13)
Diploma, year 3	54	31 (57.4)	2.03 (0.91, 4.50)
BSc, year 1	40	21 (52.5)	1.60 (0.66, 3.87)
BSc, year 2	48	20 (41.7)	0.43 (0.15, 1.29)
BSc, year 3	47	18 (38.3)	0.55 (0.20, 1.53)
BSc, year 4	45	18 (40.0)	0.57 (0.21, 1.61)
Post-diploma, post-BSc & MSc	77	59 (76.6)	2.13 (1.02, 4.45)
**Average Household Monthly Income (INR - Indian Rupees)**			
<5,000	130	62 (47.7)	1 (-)
5,001–10,000	155	80 (51.6)	1.17 (0.73, 1.87)
>10,000	136	70 (51.5)	1.16 (0.72, 1.88)
**BCG scar**			
Absent	96	41 (42.7)	1 (-)
Present	320	170 (53.1)	1.52 (0.96, 2.41)
**Past history of tuberculin testing**			
No	395	193 (48.9)	1 (-)
Yes	32	20 (62.5)	1.74 (0.83, 3.67)
**Previous TB assessment (i.e. worked-up for TB disease in the past)**			
No	401	204 (50.9)	1 (-)
Yes	29	12 (41.4)	0.68 (0.32, 1.46)

LTBI: Latent Tuberculosis Infection; TST: Tuberculin Skin Test; TB Tuberculosis.

**Table 5:** Association between measures of current and past TB exposure and TST positivity: Results of univariate logistic regression.

Variable	N	TST > = 10mm	
		Positive n (%)	Odds ratio (95% CI)
**TB exposure prior to current training program**			
Worked in health care prior to current training program			
No	356	159 (44.7)	1 (-)
Yes	80	60 (75.0)	3.72 (2.15, 6.43)
Time (years) spent in health care work after entry			
< = 1	110	54 (49.1)	1 (-)
>1 and < = 3	203	87 (42.9)	0.78 (0.49, 1.24)
>3 and < = 5	46	19 (41.3)	0.73 (0.36, 1.46)
>5	77	59 (76.6)	3.40 (1.78, 6.49)
**Non-professional or social contact with a TB patient (previously or during current training)**			
Shared a room			
No	411	207 (50.4)	1 (-)
Yes	16	10 (62.5)	1.68 (0.60, 4.71)
Shared a house			
No	405	207 (50.4)	1 (-)
Yes	27	11 (62.5)	0.66 (0.30, 1.47)
Worked together			
No	396	198 (50.0)	1 (-)
Yes	11	6 (54.5)	1.20 (0.36, 3.99)
Unknown	29	15 (51.7)	1.07 (0.50, 2.28)
Attended class			
No	383	190 (49.6)	1 (-)
Yes	18	13 (72.2)	2.64 (0.92, 7.55)
Unknown	35	16 (45.7)	0.86 (0.43, 1.71)
Socialized with			
No	369	182 (49.3)	1 (-)
Yes	23	16 (69.6)	2.35 (0.94, 5.84)
Unknown	44	21 (47.7)	0.94 (0.50, 1.75)
Any contact			
No	384	189 (49.2)	1 (-)
Yes	52	30 (57.7)	1.41 (0.78, 2.53)
**TB exposure during current training program**			
Conversational distance contact with a pulmonary TB patient			
No	116	59 (50.9)	1 (-)
Yes	200	102 (51.0)	1.03 (0.66, 1.63)
Yes but unsure if smear-positive	73	33 (45.2)	0.81 (0.45, 1.46)
Unknown	47	25 (53.2)	1.12 (0.57, 2.20)
Number of days spent working in medium or high risk wards			
0	123	67 (54.5)	1 (-)
1–90	94	55 (58.5)	1.20 (0.71, 2.04)
91–180	160	71 (44.4)	0.68 (0.43, 1.07)
>180	45	19 (42.2)	0.62 (0.32, 1.23)
Patient-days caring for active TB			
0	139	69 (49.6)	1 (-)
1–50	266	126 (47.4)	0.91 (0.61, 1.38)
>50	27	21 (77.8)	3.55 (1.35, 9.33)

LTBI: Latent Tuberculosis Infection; TST: Tuberculin Skin Test; TB: Tuberculosis.

### Multivariate analysis

In the multivariate logistic regression analysis (Table 6), TST positivity was strongly associated with time spent in health care while age at entry was no longer an important predictor. This suggests that prevalence of TST positivity increases more due to exposure to the health care environment than due to increasing age.

**Table 6:** Factors associated with TST results: multivariate logistic regression model.*

Variable	Odds ratio (95% confidence interval)
	TST> = 10mm
**Age at entry (years) (Reported age - years in health care)**	
	1.04 (0.85, 1.27)
**Time (years) spent in health care work after entry**	
	1.19 (1.10, 1.28)
**Sex**	
Male	1 (-)
Female	0.69 (0.28, 1.71)
**BCG scar**	
Absent	1 (-)
Present	1.29 (0.80, 2.08)
**Any contact**	
No	1 (-)
Yes	0.90 (0.44, 1.81)
**Number of days spent on medium risk or high risk wards**	
0	1 (-)
1–90	0.80 (0.41, 1.55)
90–180	0.61 (0.34, 1.10)
>180 (max = 310)	0.71 (0.30, 1.65)

*all variables in the model are simultaneously adjusted for each other.

### LTBI prevalence using LCA

Based on the LCA, the overall prevalence of LTBI was 47.8%, only slightly lower than the estimate based on treating TST as a perfect measure of LTBI. However, the 95% credible interval around this estimate was very wide from 17.8% to 65.6%, reflecting the uncertainty in our knowledge of the sensitivity and specificity of TST.

## Discussion

There is growing awareness about the problem of nosocomial TB and the need to protect healthcare workers in the era of multidrug-resistant (MDR) and XDR-TB. India accounts for a fifth of the world's incident TB cases and HCWs in India are constantly exposed to infectious TB patients. In this study, we established a cohort of nursing students that are highly vulnerable to TB exposure in order to study the risk due to various sources of exposure, and to gather data that might be helpful as developing countries such as India gear up to implement the new WHO TBIC policy for resource-limited settings. Our study showed a high prevalence of LTBI even in young nursing trainees - almost half the cohort was latently infected, despite the majority being under 22 years of age. A previous report among Indian health care workers found LTBI prevalence by TST to be 41.0%, but not all HCWs in this study were nurses [9].

Increasing age and increasing time spent in health care work or training should correlate with increasing cumulative TB exposure. However, TB exposure is not easy to measure in TB endemic countries. To our knowledge, this is the first study from a developing country to correlate TST with detailed and reliable exposure measurement, based on log books kept by students, of prior and current occupational TB exposure. Our analysis suggests that greater than five years spent in health care work in India is a strong risk factor for LTBI. All known exposures to a TB patient were associated with higher LTBI prevalence, although not all significantly. Work on selected high and medium risk wards did not seem to correlate with increased LTBI, but after 50 days or greater of patient-day exposure, LTBI prevalence increased markedly. This may be explained by a wide range of exposure within this category (range 50–2100). This wide range is possibly due to poor recall.

Our study had some limitations. Firstly, the TST is an imperfect test and therefore the LTBI prevalence estimate might be impacted by the test performance. Sensitization by non-tuberculous mycobacteria (NTM) is widespread in South India and could cause false-positive results. However, according to a meta-analysis [22], NTM is not a clinically important cause of false-positive TST, except in populations with a high prevalence of NTM sensitisation and a very low prevalence of TB infection. This is clearly not the case in our population in India. Digit preference noticed in our continuous TST data reflects one of the inherent problems in using the TST. To account for TST limitations, we used the latent class analysis, accounting for what is already known about TST accuracy. Interestingly, the prevalence from LCA was quite close to that obtained by using the standard cut-point analysis. This may be because BCG vaccination does not have a major impact on TST specificity in India [11], in part because BCG is given at birth in India and not repeated subsequently. There is strong evidence that BCG tends to adversely impact TST specificity when it is given after infancy and/or repeated multiple times [22].

Another obvious strategy would be to use more specific tests for LTBI, such as interferon-gamma release assays [IGRAs] [20]. We have now incorporated the QuantiFERON-TB Gold In Tube (QFT) assay in our cohort study, and after initial technical problems with quality assurance, we have successfully incorporated QFT into our annual testing protocol. Future reports will include IGRA results and provide data on conversions and reversions. Secondly, cross-sectional prevalence data have inherent limitations for determining the associations with risk factors. To overcome this, we have begun annual TST and QFT testing of all nursing students, and this will allow us to estimate the annual risk of TB infections (ARTI) and risk factors associated with new TB infection. Lastly, although we used log books to determine the TB exposure, some variables were still based on recall. Poor recall could have led to misclassification of exposure information. For example, trainees often knew that they were providing nursing care to TB patients, but did not always remember the sputum smear status of the patients.

Overall, taken together with other studies from India [4], [9], [10], [12], [15], [17], [23], our data confirm that nosocomial TB is a major, neglected problem in India. Several factors may facilitate nosocomial transmission in Indian hospitals [4], [9], [10], [12], [15], [17]. The overwhelming numbers of TB patients and repeated exposure to smear-positive TB patients is likely to be a critical factor, especially among trainees who spend a lot of time caring for sick patients in crowded wards. For example, CMC, Vellore provides care for over 5000 out-patients every day, and because CMC is a referral institute, complicated cases, including MDR and XDR-TB are managed routinely and some of them need to be hospitalized usually for short periods [24]. Repeated exposure of trainees is particularly worrisome, given the lack of adequate TBIC measures at most healthcare facilities, including CMC.

In India, students begin the undergraduate medical and nursing program at the age of 17 or 18 years. After an initial classroom-based program in basic sciences, they begin their clinical rotations and clerkships. During this phase of their training, a lot of importance is placed on bedside clinical work. Because trainees spend considerable time with patients, they are repeatedly exposed to patients with infectious TB from their first year of clinical rotations. This may explain the high prevalence of infection among them [4]. Because of their increased risk and vulnerability, young trainees and nurses, in particular, will need to be targeted for TBIC interventions.

Delays in diagnosis and initiation of treatment, and failure to separate or isolate patients with smear-positive TB from other patients are other important contributors to increased risk of transmission [1], [4]. Unnecessary or prolonged hospitalization of TB patients who could have been treated on an ambulatory basis might also contribute to the high exposure levels in hospitals. Several factors might prolong infectiousness of TB patients and thereby facilitate nosocomial transmission [1]. Poor adherence to treatment, lack of continuous drug supply, use of suboptimal treatment regimens, lack of adequate treatment support (e.g. direct observation of therapy), and insufficient treatment duration are major factors [1], [4]. High case load of TB patients in tertiary care hospitals, overcrowded out-patient clinics and inpatient wards with inadequate infection control measures, lack of awareness about TB and cough etiquette are other risk factors for increased nosocomial transmission of TB in India.

In conclusion, HCWs are essential in the fight against TB, and their health needs to be protected as well. India, with its vast human and intellectual capital, strong and rapidly growing economy and greater availability of funding, country-wide DOTS coverage, and a large, well-functioning national TB control program, is well placed to tackle this problem. While lack of resources was a genuine constraint even a decade ago, India's economy, with an annual growth rate of more than 7%, now has the resources to increase investments in TB, especially with external funding from the Global Fund to Fight AIDS, Tuberculosis and Malaria. With the recent WHO TBIC policy guidance as the reference, Indian healthcare providers (private and public) and the Revised National TB Control Programme (RNTCP) will need to begin implementing at least a minimum package of basic TB infection control measures, and enhance capacity for TBIC at the country level with adequate budget allocation. Medical schools and teaching hospitals, in particular, have to adapt and translate the TBIC policy into concrete action.
